# Accumulation of Epigenetic Noise in the Aging Corneal Epithelium and Its Possible Mechanism

**DOI:** 10.1096/fj.202500954R

**Published:** 2025-06-09

**Authors:** Galina Dvoriantchikova, Michelle Fleishaker, Dmitry Ivanov

**Affiliations:** ^1^ Bascom Palmer Eye Institute, Department of Ophthalmology University of Miami Miller School of Medicine Miami Florida USA; ^2^ Bascom Palmer Eye Institute, Department of Ophthalmology, Department of Microbiology and Immunology University of Miami Miller School of Medicine Miami Florida USA

**Keywords:** aging, cornea, corneal epithelium, DNA Hydroxymethylation, DNA methylation, epigenetic noise, epigenetics, limbal epithelial stem cells, TET enzymes

## Abstract

The cornea is the eye's “window” and plays an important role in vision. Aging has a substantial impact on corneal function by reducing the ability of corneal cells to protect the eye, refract light, and repair itself. In this study, we investigated DNA methylation patterns and the activity of the DNMT and TET families, which are responsible for shaping these patterns, in the aging corneal epithelium. To this end, we used corneal epithelial cell sheets detached from the corneas of 2‐ and 14‐month‐old mice to study gene expression, DNA methylation, and DNA hydroxymethylation. We detected significant changes in gene expression in aging corneal epithelial cells. Our data indicate that aging leads to significant changes in the methylation of individual cytosines and large DNA regions, which were similar to those shown for other aging tissues. We observed reduced expression of genes from the DNMT and TET families and reduced DNA hydroxymethylation levels in the corneal epithelium of 14‐month‐old mice compared to 2‐month‐old mice. These data indicate that the activity of TET enzymes is reduced in the corneal epithelium during aging. Thus, we found an accumulation of epigenetic noise in the aging corneal epithelium, manifested by increases and decreases in DNA methylation levels, which may be caused by decreased activity of TET enzymes. We propose that the observed age‐related changes in the corneal epithelium reflect epigenetic changes occurring in the limbal epithelial stem cells.

## Graphical abstract

We found an accumulation of epigenetic noise in the aging corneal epithelium, manifested by increases and decreases in DNA methylation levels. Our findings suggest that these changes occur due to reduced activity of TET enzymes. We assume that the age‐related changes in DNA methylation patterns and the activity of TET enzymes reflect the epigenetic state of the limbal epithelial stem cells, since corneal epithelial cells are short‐lived cells.

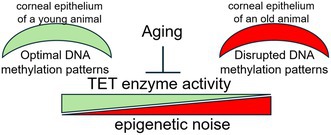

## 1 Introduction

A healthy cornea is vital for clear vision because it accounts for two‐thirds of the eye's total focusing power, while the lens accounts for only one‐third [1, 2, 3, 4]. Therefore, disturbances in corneal function result in various diseases that, if left untreated, lead to blindness [5, 6, 7, 8]. One of the important conditions for maintaining normal corneal function is the constant process of replenishment of corneal epithelial cells due to the activity of the limbal epithelial stem cells (LESCs) [1, 2, 8]. LESC deficiency due to injury or disease results in deleterious effects on wound healing and surface integrity, neovascularization, chronic inflammation, and recurrent erosions, leading to painful vision loss [8]. Thus, maintaining the normal state of LESCs throughout life is important for preserving vision.

The accumulation of damage in cells is the driving factor behind their aging [9]. In addition to damage to proteins, lipids, and DNA, changes in epigenetic patterns in cells with age are also considered a form of damage [9, 10]. As each cell type develops, epigenetic patterns are formed that support the expression of important genes and prevent the expression of unnecessary genes [11]. Changes in these epigenetic patterns with age can lead to disruption of optimal gene expression, leading to impaired cell function or even cell death [9, 10]. DNA methylation, a stable epigenetic mark, plays a special role in this process since it can be inherited through multiple cell divisions [12, 13]. Changes in DNA methylation patterns, once occurring in a cell, can persist in it and its progeny. DNA methylation patterns are established by the balanced activity of two families: the DNA methyltransferase (DNMT) family is responsible for the methylation of cytosines in DNA and maintaining their methylated state during cell proliferation, while the ten‐eleven translocation (TET) family is responsible for DNA demethylation [12, 14, 15]. Stresses encountered during an organism's life can disrupt the balance of activity of these families, leading to changes in DNA methylation patterns. Changes in methylation of several cytosines are not dangerous, but the accumulation of such cytosines with age can lead to a decrease in the expression of important genes and to an increase in the expression of genes undesirable for cell function. Such cells and their corresponding tissues cannot function properly, which is characteristic of old tissue [9, 10]. The accumulation of cytosines whose methylation changes randomly with age is known as epigenetic noise. The objective of this study was to investigate whether aging leads to the accumulation of epigenetic noise in the corneal epithelium and LESCs.

LESCs are located in the limbal stem cell niche (LSCN) [1, 2, 8]. The LSCN is made up of a variety of cells that support the function and survival of LESCs [1, 2, 8]. The LSCN, in turn, is located in the corneal limbus, which is a highly innervated and vascularized region between the cornea, conjunctiva, and sclera [1, 2, 8]. The small size of the limbus and the significant diversity of cells within it do not allow for the isolation of LESCs from it in a quantity and purity that is sufficient for their epigenetic analysis. However, because LESC‐derived corneal epithelial cells are completely renewed within 1–2 weeks, this is not enough time for corneal epithelial cells to accumulate epigenetic noise [16, 17, 18]. Therefore, the changes in DNA methylation patterns that we can observe in the corneal epithelium reflect age‐related changes in LESCs. The large number of corneal epithelial cells in the cornea can provide sufficient material to study the accumulation of epigenetic noise in aging LESCs.

## 2 Materials and Methods

### 2.1 Animals

All procedures were executed in compliance with the NIH Guide for the Care and Use of Laboratory Animals, the ARVO statement for the Use of Animals in Ophthalmic and Vision Research, and according to the University of Miami IACUC approved protocol. C57BL/6J mice were obtained from the Jackson Laboratory (Bar Harbor, ME, USA; #000664). We used 2‐ and 14‐month‐old male and female mice to address sex as a biological variable. The animals were housed under standard conditions of humidity and temperature, were given free access to food and water, and had a 12‐h light to dark cycle.

### 2.2 Corneal Epithelial Cell Sheet Removal

To collect corneal epithelial cell sheet, each individual cornea was placed in 1 mL of HBSS without Ca2+ and Mg2+ containing Dispase II (2.4 U/mL, 04942078001, MilliporeSigma, Burlington, MA, USA) for 1 h at 37C. At the end of the enzyme treatment, the corneal epithelial cell sheet was removed by gentle teasing with a pair of forceps [19]. The collected corneal epithelial cell sheet was rinsed 3 times with 1x PBS and used for further applications.

### 2.3 Immunohistochemistry

Eyes were enucleated and fixed with 4% paraformaldehyde (PFA) in phosphate‐buffered saline (PBS) overnight at 4°C. The next day, the fixed eyes were transferred to 30% sucrose in PBS, where they were kept for 24 h. The eyes were then cryo‐embedded in OCT and cryo‐sectioned at 10 μm thickness. The sections were permeabilized with 0.3% Triton X‐100/PBS for 1 h, washed with PBS, and then blocked in a buffer containing 5% donkey serum, 2% BSA, and 0.15% Tween‐20 in PBS. The sections were incubated overnight with Krt12 antibody (1:200, MA5‐42701, ThermoFisher Scientific, Waltham, MA, USA) at 4°C. The next day, the sections were washed with PBS and incubated with donkey anti‐rabbit secondary fluorescent antibodies. The sections were then washed with PBS and 0.15% Tween‐20. DAPI was added during sample washing to detect cell nuclei. Finally, the sections were mounted with mounting media and coverslip. Control sections were incubated without primary antibodies. Imaging was performed with the Leica STELLARIS confocal microscope (Leica Microsystems, US).

### 2.4 RNA and DNA Purification; Quality and Quantity Control

Total RNA was isolated from corneal epithelial cell sheets using RNeasy Plus Mini Kit (#74134, Qiagen, Hilden, Germany). RNA quality and quantity were measured by the NanoDrop One spectrophotometer and Qubit 4 Fluorometer (ThermoFisher Scientific, Waltham, MA, USA). We used the 2100 Bioanalyzer Instrument (Agilent Technologies, Santa Clara, CA, USA) to assess RNA integrity. The RNA samples, which had a RIN score of 8 or higher, were used to prepare RNA‐seq libraries. Genomic DNA (gDNA) was isolated from corneal epithelial cell sheets using the DNeasy Blood and Tissue Kit (#69504, Qiagen, Hilden, Germany). The gDNA samples were quantified using the Qubit High Sensitivity dsDNA kit and Qubit 4 (ThermoFisher Scientific, Waltham, MA, USA). The quality of the gDNA samples was checked on the 4200 TapeStation system using the Genomic DNA ScreenTape Kit (Agilent Technologies, Santa Clara, CA, USA).

### 2.5 RNA‐Seq Library Preparation, Sequencing, Data Analysis

The RNA‐seq libraries were prepared using the Illumina Stranded mRNA Prep Kit (#20040532, Illumina, San Diego, CA, USA) and IDT for Illumina RNA UD Indexes Set A (#20040553, Illumina, San Diego, CA, USA) according to the manufacturer's instructions. The quality/quantity of the RNA‐seq libraries was evaluated using Qubit 4, NanoDrop One, and the 2100 Bioanalyzer Instrument. The RNA‐seq libraries were multiplexed and sequenced on the Illumina Novaseq 6000 with a 2 × 150 paired end (PE) configuration. RNA‐seq raw FASTQ data were analyzed using the STAR software, HTseq package, and edgeR and ViDGER Bioconductor packages [20, 21, 22]. The gene ontology (GO) analysis was performed using iDEP 2.01 (pathway analysis method: GAGE; pathway significance cutoff: FDR < 0.1) [23].

### 2.6 Whole Genome Bisulfite Sequencing (WGBS) Library Preparation and Data Analysis

WGBS libraries were prepared using xGen Methyl‐Seq DNA Library Prep Kit (#10009860, Integrated DNA Technologies, Coralville, IA, USA) according to manufacturer's instructions. WGBS libraries were quantified using Qubit 4, and sizes were evaluated using the 4200 TapeStation system. The libraries were sequenced on the NextSeq2000 to generate PE‐150 reads. We used FastQC (v0.11.8) to assess the overall quality of each sequenced sample. We used TrimGalore (v0.4.5) with the following parameters: –adapter AGATCGGAAGAGC ‐e 0.1 –stringency 6 –length 20 –nextseq 20 –three_prime_clip_R1 10 –clip_R2 10. Bismark Bisulfite Read Mapper and Methylation Caller, and GENCODE M25 (GRCm38.p6) *Mus musculus* reference (mm10) genome were used to align trimmed paired‐end reads, to determine the methylation state of cytosines, and to compute the percentage of methylation [24]. To calculate the average X coverage, we used the DSS Bioconductor package [25]. Our results indicate that the average base coverage of the mouse genome was 20X for CE2m_1, 16X for CE2m_2, 21X for CE2m_3, 20X for CE14m_1, 22X for CE14m_2, 18X for CE14m_3. To perform CpG methylation clustering and CpG methylation PCA analysis, and to detect differentially methylated sites (DMSs), we used the methylKit Bioconductor package and our DNA methylation data from which X, Y, and M chromosomes were excluded to eliminate the effect of sex as a biological variable [26]. To detect differentially methylated regions (DMRs), we used dmrseq Bioconductor package and our DNA methylation data that also did not contain X, Y, and M chromosomes [27]. The EnrichedHeatmap Bioconductor package was used to visualize the enrichment of methylated regions over transcription start sites [28]. Annotation was accomplished using the annotatr Bioconductor package [29]. GREAT (Genomic Regions Enrichment of Annotations Tool) and a collection of databases HAGR were used to assess whether DMRs are near genes with known age‐related functions [30, 31, 32, 33].

### 2.7 Hydroxymethylated DNA Immunoprecipitation and Sequencing (hMeDIP‐Seq) Library Preparation

To prepare hMeDIP‐seq libraries, 1 μg of gDNA was fragmented to 350 bp (Covaris M220, Covaris, Woburn, MA, USA). The fragmented DNA was cleaned, denatured, and resuspended in immunoprecipitation buffer. 2 μg of a 5hmC‐specific antibody (#39791, Active Motif, Carlsbad, CA, USA) was added for immunoprecipitation (IP) overnight at 4C. 10% of the DNA being used in the IP reaction was saved for use as Input (IN) DNA. Protein G magnetic beads were used to perform the pull‐down of 5hmC‐enriched fragments. The 5hmC‐enriched DNA fragments (IP) were then released from the antibody by digestion with Proteinase K. After cleanup with AMPure XP beads, the 5hmC‐enriched DNA fragments (IP) and Input (IN) DNA were used to prepare libraries using the NEBNext Ultra II DNA Library Prep (E7103S, NEB, Ipswich, MA, USA). The hMeDIP‐seq libraries were quantified using Qubit 4, and sizes were evaluated using the 4200 TapeStation system. The libraries were finally pooled and submitted for sequencing on the Illumina NovaSeq 6000.

### 2.8 hMeDIP‐Seq Data Analysis

The FastQC package was used to assess the overall quality of each FASTQ file. We used TrimGalore (v0.4.5) with the following parameters: –adapter AGATCGGAAGAGC ‐e 0.1 –stringency 6 –length 20 –nextseq 20. Trimmed reads were aligned to *Mus musculus* reference (mm10) genome with Bowtie2 using default parameters [34]. Peaks were called with MACS2 using default parameters [35]. To eliminate the effect of sex as a biological variable, we used MACS2 DNA hydroxymethylation data from which X, Y, and M chromosomes were excluded. To detect differentially hydroxymethylated regions, we used the DiffBind Bioconductor package according to the manual [36]. Annotations were accomplished using the annotatr package.

### 2.9 Statistical Analysis

We used the unpaired Student's t‐test for experiments containing one variable. *P‐values* equal to or less than 0.05 were considered statistically significant. Generation and analysis of next‐generation sequencing (NGS) data were carried out in‐house according to ENCODE standards and pipelines with *n* = 3 (2 m) and *n* = 3 (14 m) for RNA‐seq, WGBS, and hMeDIP‐seq. The details of this analysis are given above.

## 3 Results

### 3.1 Gene Expression Changes as the Corneal Epithelium Ages

**FIGURE 1:**
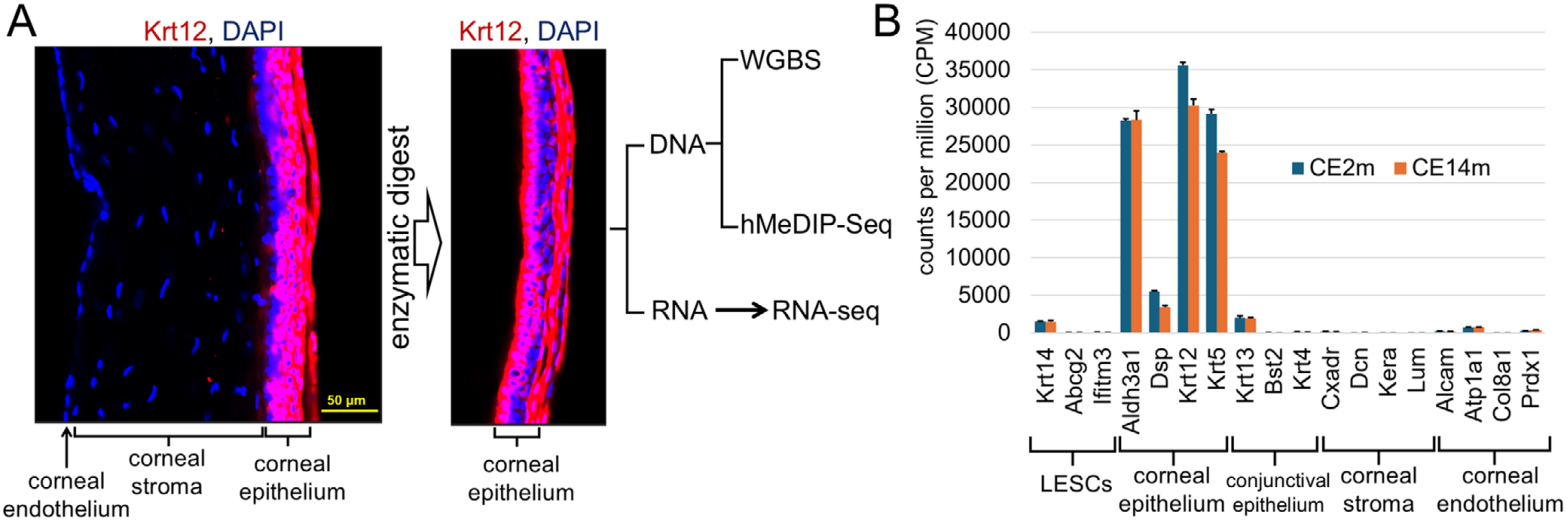
Corneal epithelial cell sheets were used to study the accumulation of epigenetic noise during aging. (A) Corneal epithelial cell sheets detached from corneas of 2‐ and 14‐month‐old mice were used for RNA and DNA purification. To prepare one RNA sample, corneal epithelial cell sheets detached from corneas of left eyes of 3 mice were pooled and used for RNA purification. To prepare one DNA sample, corneal epithelial cell sheets detached from corneas of right eyes of the same 3 mice were pooled and used for DNA purification. Animals of the same sex (males or females) were used for each sample preparation. We applied RNA‐seq, WGBS, and hMeDIP‐seq approaches to examine gene expression, DNA methylation patterns, and TET enzyme activity in these samples. (B) High expression of corneal epithelial cell markers and insignificant expression of markers of other corneal cell types in the corneal epithelial cell sheets indicate the purity and specificity of the samples we collected.

**FIGURE 2:**
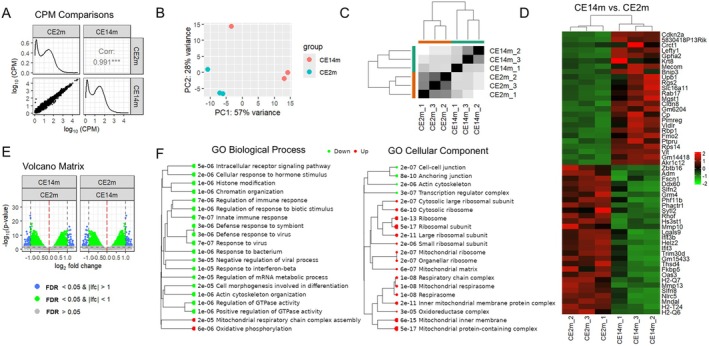
Gene expression in the corneal epithelium of old mice differs from that in young mice. (A) RNA‐seq analysis shows significant differences in corneal epithelial cell gene expression between 2‐ and 14‐month‐old mice. (B–D) Principal component analysis (PCA, B) and heatmaps (C, D) indicate a clustering of RNA samples according to the age of the animals from which they were collected. (E) The volcano plot shows that the number of genes that are up‐ and downregulated is approximately the same between young and old mice (lfc—logarithmic fold change). (F) The GO enrichment analysis was performed to identify important biological processes and cellular components during aging.

In our study, we used 2‐ and 14‐month‐old mice. Viable, large, and intact corneal epithelial cell sheets were detached from corneas using Dispase II (Figure 1A) [19]. We pooled three corneal epithelial cell sheets to obtain a sample, which was subsequently used for either DNA or RNA purification (Figure 1A). We used the RNA‐seq analysis to study changes in gene expression caused by aging. To this end, RNA isolated from our samples was used to prepare RNA‐seq libraries (*n* = 3 for 2‐month‐old [CE2m] and *n* = 3 for 14‐month‐old [CE14m] mice). To analyze RNA‐seq data, we used the edgeR Bioconductor package [22]. We first assessed the possible contamination of our samples with other corneal cell types. We found that only corneal epithelium markers were highly expressed (Figure 1B) [37, 38, 39]. The differential gene expression analysis shows a distinction in gene expression between CE2m and CE14m samples, as indicated by the value of the correlation coefficient (0.991), principal component analysis (PCA), and heatmaps (Figure 2A–D). We found that the expression of 661 genes was more than 1.5X higher in CE14m compared to CE2m samples, while the expression of 437 genes was more than 1.5X higher in CE2m compared to CE14m samples (FDR < 0.05, Figure 2E, File S1). Many genes whose expression was increased in old (CE14m) animals were involved in mitochondrial respiratory chain complex assembly (FDR = 2·10^−5^) and oxidative phosphorylation (FDR = 6·10^−6^) (Figure 2F). The expression of genes whose products are structural components of mitochondria was also increased in old (CE14m) mice. At the same time, the expression of genes involved in chromatin organization (FDR = 1·10^−6^), innate immune response (FDR = 7·10^−7^), response to virus (FDR = 7·10^−7^), response to bacterium (FDR = 1·10^−6^), and cell morphogenesis involved in differentiation (FDR = 2·10^−5^) was reduced in old mice (Figure 2F). Our results suggest that mitochondrial respiration and the activity of systems necessary for defense against pathogens and for maintaining the chromatin structure are altered in the aging corneal epithelium.

### 3.2 Aging Alters the DNA Methylation Pattern in the Corneal Epithelium

**FIGURE 3:**
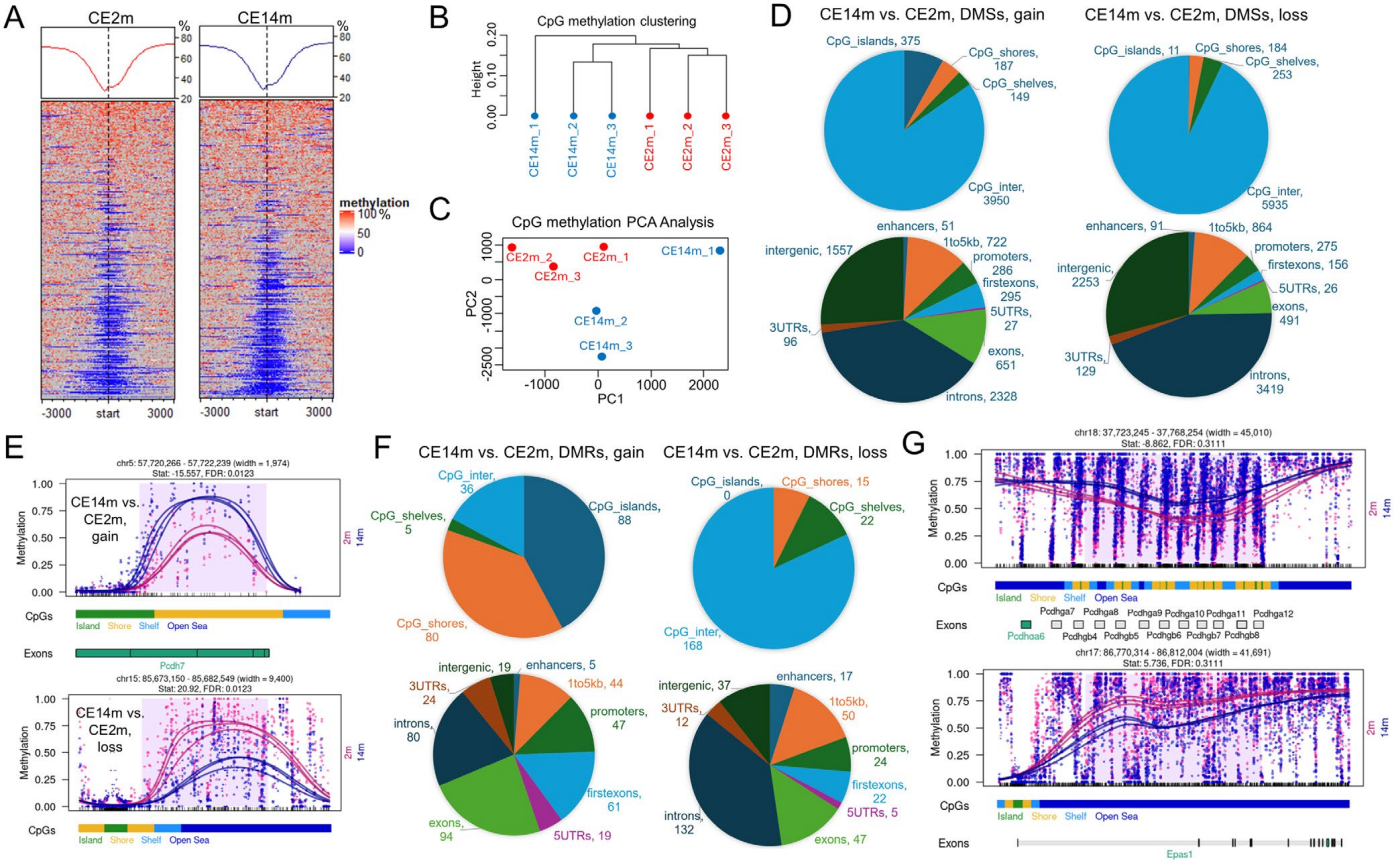
DNA methylation patterns in short‐lived corneal epithelial cells differ greatly between young and old mice, implicating LESCs as the primary source of these changes. (A) To visualize the enrichment of methylated regions over transcription start sites (TSS) in the corneal epithelium of young (CE2m) and old (CE14m) mice, we used the EnrichedHeatmap Bioconductor package. The horizontal axis in the panel corresponds to the position of CpGs surrounding a TSS of a gene (− 3000, + 3000 bp). The vertical axis corresponds to all known genes in the mouse genome. The methylation % of each CpG is highlighted in color. The graph at the top is the average CpG methylation % at a given position relative to a TSS (− 3000, + 3000 bp; horizontal axis) across all genes (vertical axis). (B, C) CpG methylation clustering (B, Ward's method was applied) and CpG methylation PCA analysis (C) were carried out using the methylKit package. (D) We annotated differentially methylated sites (DMSs) and counted their number in the annotated regions. DMSs where the difference in methylation level between the CE2m and CE14m differed by 10% or more were used in the analysis (gain—the level of methylation is increased in CE14m compared to CE2m; loss—the methylation level is reduced in CE14m). (E) The panel provides examples of statistically significant differentially methylated regions (DMRs) in CE14m vs. CE2m with increased and decreased DNA methylation levels. (F) We counted the number of DMRs depending on their position in the annotated regions. We used DMRs in which the difference in DNA methylation levels was 10% or more. (G) The panel represents examples of large‐scale methylation blocks of genomic DNA containing multiple DMRs.

To study DNA methylation patterns in the corneal epithelium of the young (2 month) and old (14 month) mice at single nucleotide resolution, we used WGBS. To this end, DNA isolated from our samples was used to construct 6 WGBS libraries (*n* = 3 for 2‐month‐old [CE2m] and *n* = 3 for 14‐month‐old [CE14m] mice). We used the Bismark package to calculate the percentage of methylation of each cytosine in the CpG context [24]. Our results indicate that average methylation patterns near the transcription start sites (TSS) do not differ in the corneal epithelium of young and old animals (Figure 3A). However, considering the percentage of methylation of each cytosine in the genome, CpG methylation clustering and CpG methylation PCA analysis indicate that DNA methylation patterns differ in the corneal epithelium of 2‐ and 14‐month‐old animals (Figure 3B,C). These patterns are similar in young (CE2m) mice, yet more diverse in old (CE14m) mice (Figure 3B,C). Using the methylKit Bioconductor package, we found 11,511 differentially methylated sites (DMSs, FDR < 0.05, File S2) [26]. 5118 DMSs showed an increase in methylation levels, with 4661 DMSs showing an increase of more than 10% in CE14m; 6393 DMSs showed an increase in methylation levels, with 4651 DMSs showing an increase of more than 10% in CE2m (File S2). These sites are largely located in introns and exons of genes, as well as in intergenic regions (Figure 3D). Because changes in methylation levels of many CpG sites in a small DNA region can have a larger impact on gene activity, detection of these regions is of great importance. To this end, we used the dmrseq Bioconductor package to detect differentially methylated regions (DMRs) in CE2m vs. CE14m [27]. We found 336 statistically significant DMRs, among which 315 regions had average DNA methylation levels that differed by more than 10% between CE2m and CE14m (FDR < 0.05, File S2). As with DMSs, the number of DMRs with increased and decreased DNA methylation levels was approximately the same (Figure 3E, File S2). Most DMRs with increased DNA methylation levels in old animals were found in promoters, exons, and introns of genes and in CpG islands (Figure 3F, File S2). Meanwhile, in old animals, the level of DNA methylation was reduced in many DMRs located in exons and introns of genes, as well as in intergenic regions (Figure 3F, File S2). Of note, the changes we discovered in the level of DNA methylation may affect large regions that contain many genes (Figure 3G). These regions include those in which the clustered protocadherins (PCDHs) are located (Figure 3G). It has been previously shown that the level of methylation in these gene clusters changes significantly during aging [40, 41]. Still, we did not observe a strong correlation between individual gene expression changes and proximal methylation changes (File S2). We can attribute this to the fact that most changes in DNA methylation are observed in introns and intergenic regions. However, changes in DNA methylation can affect not only the activity of the genes in which these changes occurred, but also nearby genes that may be involved in the aging process. We used the Genomic Regions Enrichment of Annotations Tool (GREAT) and a collection of databases HAGR to assess whether the DMRs we identified are near genes with known age‐related functions [30, 31, 32, 33]. We found more than 500 genes located near the DMRs (Supplementary Figure S1, File S2). Seventy‐two of these genes are involved in the aging process (File S2). The expression of a number of these age‐related genes changed more than twofold (*Irs2*, logFC = −1.02, FDR = 0.004; *Cdkn2a*, logFC = 2.75, FDR = 0.003; *Rgs2*, logFC = 1.62, FDR = 0.02; *Ptpn22*, logFC = 1.15, FDR = 0.004; *Adamtsl1*, logFC = −1.22, FDR = 0.03). These findings suggest that changes in DNA methylation may influence the aging of the corneal epithelium by affecting the activity of nearby age‐related genes. Finally, our results show that aging leads to changes in DNA methylation patterns in the corneal epithelium, which are equally associated with both an increase and a decrease in the level of DNA methylation.

### 3.3 TET Enzyme Activity Is Reduced in the Corneal Epithelium of Old Animals

**FIGURE 4:**
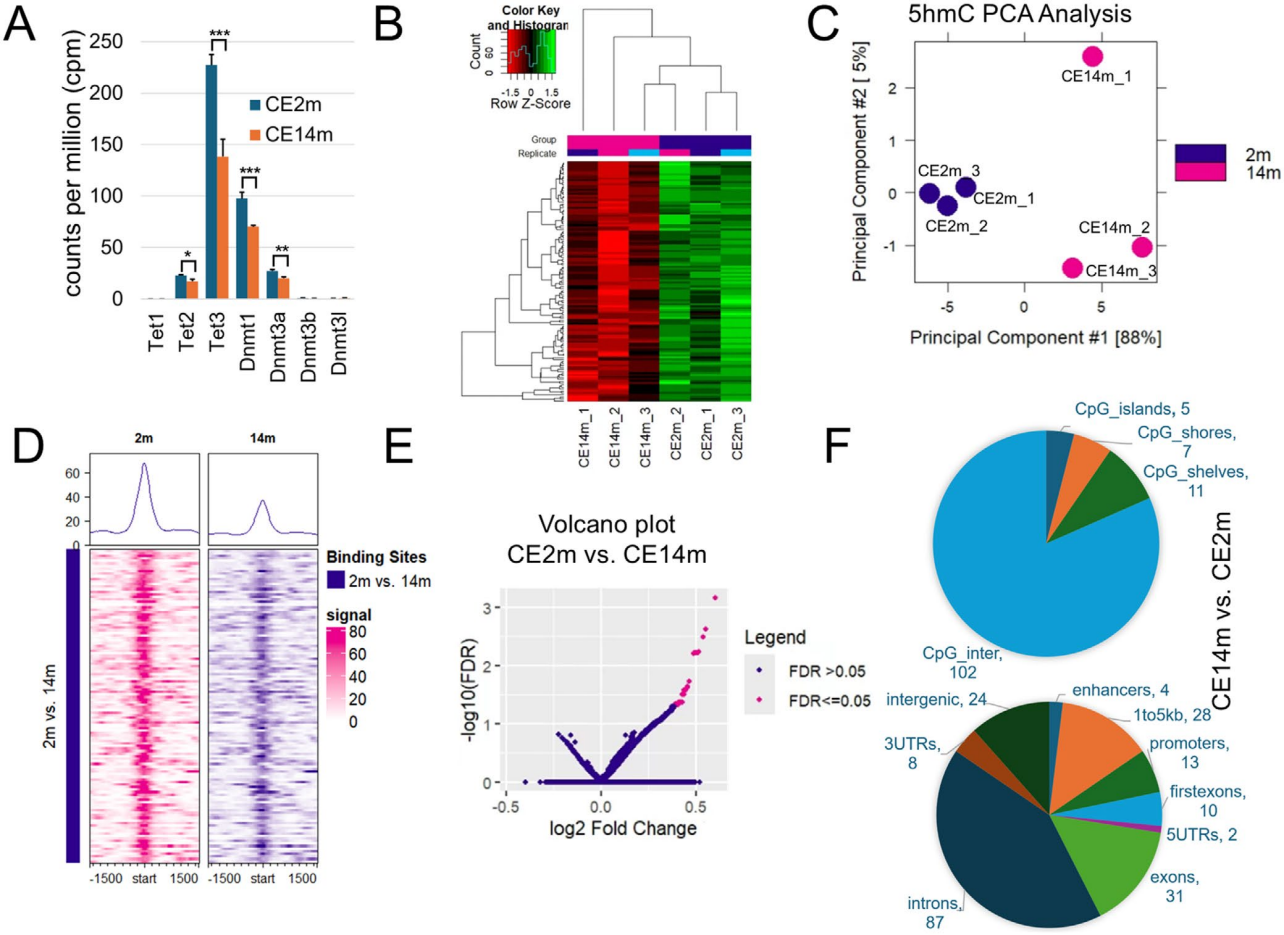
Aging leads to an imbalance in the activity of pathways responsible for establishing and maintaining the DNA methylation pattern in corneal epithelial cells. (A) The expression of genes belonging to the DNMT and TET families was determined in the corneal epithelium using RNA‐seq analysis (*P‐value < 0.05, **P‐value < 0.01, ***P‐value < 0.001). (B, C) hMeDIP‐seq is a variation of ChIP‐seq where antibody recognizing hydroxymethylated cytosines (5hmC‐specific antibody) is used. In addition, hMeDIP‐seq uses DNA rather than chromatin. The panel (B) exhibits the binding affinities and clustering for differentially bound sites, which were identified by the hMeDIP‐seq using the DiffBind Bioconductor package. To this end, the normalized read counts were row scaled, and green/red heatmap colors were used. 5hmC PCA plot (C) was generated from hMeDIP‐seq data using the DiffBind Bioconductor package. (D) DiffBind plot is generated on the hMeDIP‐seq binding data of 5hmC‐specific antibodies to DNA. The vertical axis shows all identified hMeDIP‐seq peaks. The horizontal axis reflects how strongly (signal) antibodies bind to the DNA around a peak (−1500, 1500 bp). The signal strength is determined by the number of reads corresponding to the DNA region around a peak. The graph at the top corresponds to the average signals obtained across all peaks. DiffBind generated plot with sample groups merged shows that DNA hydroxymethylation levels are reduced in the corneal epithelium of old (14 m) compared to young (2 m) animals. There are no regions in which these levels are increased in old animals. (E) The volcano plot shows that regions with increased levels of DNA hydroxymethylation exist only in the corneal epithelium of young animals (red dots). (F) Regions with reduced DNA hydroxymethylation levels in old animals were annotated.

Balanced activity of the DNMT and TET families is necessary to establish and maintain optimal DNA methylation patterns in cells [12, 14, 15]. Because DNA methylation patterns in the corneal epithelium of old mice (CE14m) differed from those of young animals (CE2m), we hypothesized that changes in the activity of the DNMT and TET families led to these changes. The DNMT family includes *Dnmt1*, *Dnmt3a*, *Dnmt3b*, and *Dnmt3l* genes. *Dnmt3a* and *Dnmt3b* are responsible for *de novo* DNA methylation, while *Dnmt1* is responsible for the maintenance of DNA methylation patterns in dividing cells [12, 14, 15]. The TET family includes *Tet1*, *Tet2*, and *Tet3*. The enzymes encoded by these genes are responsible for DNA demethylation and for maintaining the unmethylated state of important genes [12, 14, 15]. Reduced activity of TET enzymes can lead to increased methylation of some DNA regions and decreased methylation of other DNA regions [15, 42, 43]. Using our RNA‐seq data, we found that the gene expression of the DNMT and TET families is reduced in the corneal epithelium of old mice (Figure 4A). However, the strongest reduction is observed for *Tet3* (logFC = −0.72, FDR = 0.01). Since *Tet3* has the highest expression in the TET family, a significant decrease in its expression may lead to a decrease in the activity of TET enzymes in the corneal epithelium of old animals (Figure 4A). The intermediate products of TET enzyme activity during DNA demethylation are hydroxymethylated cytosines (5hmC). By determining the levels of these modified nucleotides in DNA, we can assess the activity of TET enzymes in a tissue. To this end, we used the hMeDIP‐seq approach to detect regions of DNA enriched with 5hmC nucleotides. DNA isolated from our samples was used to prepare 6 hMeDIP‐seq libraries (*n* = 3 for CE2m and *n* = 3 for CE14m). We used the MACS2 and DiffBind packages to analyze our data and to detect DNA regions that differ in levels of 5hmC between young (CE2m) and old (CE14m) mice. Our findings indicate that DNA hydroxymethylation patterns in the corneal epithelium are similar in young animals, but more diverse in old animals (Figure 4B,C). We found 122 regions in which the level of DNA hydroxymethylation differed statistically significantly between young and old animals (FDR < 0.05, File S3). In all these regions, the level of DNA hydroxymethylation was reduced in the corneal epithelium of old mice compared to young mice (Figure 4D,E). These changes in DNA hydroxymethylation levels mainly affect exons and introns of genes and intergenic regions (Figure 4F). Our body of evidence indicates that the activity of TET enzymes is reduced in the corneal epithelium of old animals, which may account for changes in DNA methylation patterns during corneal aging.

## 4 Discussion

Aging causes structural and functional changes in the cornea, which can lead to deterioration of vision [44, 45, 46]. These changes affect all cell layers of the cornea, including the corneal epithelium [44, 45, 46]. It is widely recognized that the process of random accumulation of damage, which includes epigenetic noise, is responsible for age‐related structural and functional changes in a tissue [9, 10]. In our study, we found an accumulation of epigenetic noise, manifested in an increase and decrease in DNA methylation levels in different regions of the genome of the aging corneal epithelium. We found decreased expression of genes of the DNMT and TET families in the aging corneal epithelium. The level of DNA hydroxymethylation was decreased in the aging corneal epithelium as well, indicating that the activity of TET enzymes in the corneal epithelium decreases with age. Since corneal epithelial cells are short‐lived cells, we propose that the observed age‐related changes in DNA methylation patterns and the activity of TET enzymes correspond to epigenetic changes in aging LESCs.

Corneal epithelial cells are short‐lived cells, whose population is constantly replenished by LESC activity [1, 2, 8]. Hence, changes in corneal epithelial cells during aging reflect changes occurring in LESCs. Since methylated DNA cytosines are a stable epigenetic mark, we can detect changes that accumulate in LESCs during aging by analyzing DNA methylation patterns in the corneal epithelium of young and old animals. Changes in DNA methylation patterns have been shown to contribute to retinal aging and the development of age‐related diseases such as glaucoma [10]. At the same time, restoring youthful DNA methylation patterns by increasing the activity of TET enzymes improves vision and reduces retinal damage in glaucoma [10]. Furthermore, the study of Hernando–Herraez showed the accumulation of stochastic DNA methylation changes in promoters of aging mouse muscle stem cells, which were associated with the degradation of coherent transcriptional networks [47]. We found similar changes in the corneal epithelium and link them to changes in the DNA methylation patterns of aging LESCs. Reduced activity of TET enzymes during aging may play a significant role in altering the DNA methylation patterns in LESCs. However, it should be noted that our findings regarding changes in DNA methylation patterns in aging LESCs are indirect, which is a limitation of the study. High‐throughput single‐cell DNA methylation profiling of young and old LESCs would overcome this limitation. While this technology is still new and very expensive, we plan to use it in future studies of LESC and corneal epithelial aging.

Impaired function and decreased numbers of adult stem cells due to accumulation of damage, including epigenetic noise, are among the driving factors of non‐neuronal tissue aging [48]. The inability of aging adult stem cells to generate the required number of normally functioning cells can lead to diminished tissue function, which is manifested in an aging phenotype [48]. This is true for the cornea as well [4, 8, 44, 45]. Thus, understanding the mechanisms that lead to changes in DNA methylation patterns in LESCs and the corneal epithelium during aging is important for creating medications that will slow down or reverse this process. Our results provide a promising lead. We found that aging has a significant impact on the activity of TET enzymes in the corneal epithelium. There is a growing body of evidence that decreased TET enzyme activity leads to changes in DNA methylation patterns that include both increases and decreases in methylation levels of individual CpG sites and regions [15, 42, 43]. Thus, the reduced activity of TET enzymes that we found may explain the change in DNA methylation patterns that we observed. The activity of TET enzymes depends on the oxygen and glucose levels in a cell [14, 15]. Our RNA‐seq results suggest altered mitochondrial respiration in old animals, which may lead to increased oxygen and glucose consumption. TET enzymes in such conditions may experience a lack of oxygen and glucose, affecting their optimal activity and contributing to changes in DNA methylation patterns. The contribution of reduced expression of DNMT family to the hypomethylation side of the epigenetic noise should also not be excluded (Figure 4A). It was shown that reduced DNMT activity, whether due to mutations or decreased expression, results in DNA hypomethylation [49]. Overall, a disrupted optimal balance of activity of TET and DNMT families during aging may lead to significant changes in DNA methylation patterns. However, changes in DNA methylation patterns are only possible in aging adult stem cells such as LESCs and muscle stem cells, or long‐lived cells like neurons, but not in short‐lived corneal epithelial cells. Thus, we can propose that DNA methylation patterns first change in LESCs and then, by extension, in the corneal epithelial cells.

In conclusion, we found that DNA methylation patterns change significantly in the corneal epithelium during aging. Our results suggest that these changes occur due to an imbalance in the activity of DNMT and TET families: we have shown that TET enzyme activity is reduced in the corneal epithelium of old mice. Considering that corneal epithelial cells are short‐lived cells, we propose that our results reflect epigenetic changes occurring in aging LESCs. Given that there may be interspecies differences in the aging process, it would be important to investigate the occurrence of epigenetic noise in human LESCs and corneal epithelium during aging in the future.

## Author Contributions

D.I. conceived and supervised the project. G.D., M.F., and D.I. performed the experiments and the data analyses. G.D., M.F., and D.I. assisted with the bioinformatic analysis. G.D., M.F., and D.I. assisted with the research design, data interpretation, manuscript writing, and editing. All authors have read and agreed to the published version of the manuscript.

## Conflicts of Interest

The authors declare no conflicts of interest.

## Supporting information


**Supporting Information S1.** Results of the RNA‐seq analysis

**Supporting Information S2.** WGBS data and analysis

**Supporting Information S3.** Results of the hMeDIP‐seq analysis

**Supplementary Figure S1.** The integrative analysis using GREAT revealed many DMRs that may influence the activity of nearby genes. (A) Most of the DMRs are located near two genes whose activity they could influence. Many DMRs where methylation is increased in the corneal epithelium of old mice (CE14m) are located closer to the transcription start site (TSS) of these genes. (B) A comprehensive analysis of the identified genes was conducted to identify the biological processes and diseases in which they are involved.

## Data Availability

Stored in repository.
