# Supplementary figures and images for: Accumulation of Epigenetic Noise in the Aging Corneal Epithelium and Its Possible Mechanism
Source: FASEB J. 2025 Jun 9;39(11):e70699. doi: 10.1096/fj.202500954R (PMC12147993; doi:10.1096/fj.202500954R)

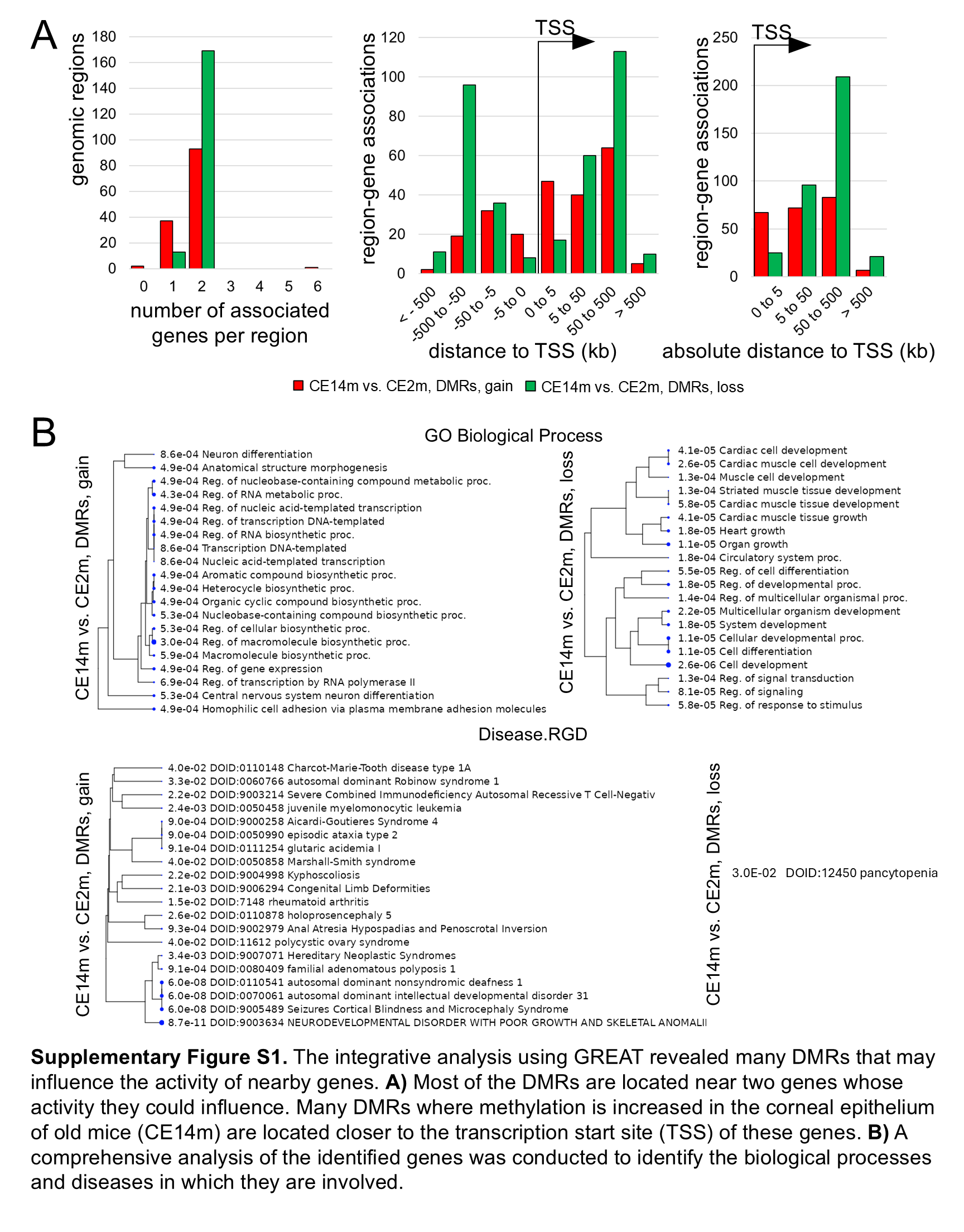

Supplement: Supplementary file 4 — Supplementary Figure S1. The integrative analysis using GREAT revealed many DMRs that may influence the activity of nearby genes. (A) Most of the DMRs are located near two genes whose activity they could influence. Many DMRs where methylation is increased in the corneal epithelium of old mice (CE14m) are located closer to the transcription start site (TSS) of these genes. (B) A comprehensive analysis of the identified genes was conducted to identify the biological processes and diseases in which they are involved. [file FSB2-39-e70699-s003.tif]
